# Complete atrioventricular conduction system pacing in dextrocardia—a case report

**DOI:** 10.1093/ehjcr/ytag267

**Published:** 2026-04-15

**Authors:** Catalin Pestrea, Ecaterina Cicala, Maria Pana, Florin Ortan

**Affiliations:** Department of Interventional Cardiology, Brasov County Clinical Emergency Hospital, Brasov 500326, Romania; Department of Interventional Cardiology, Brasov County Clinical Emergency Hospital, Brasov 500326, Romania; Department of Interventional Cardiology, Brasov County Clinical Emergency Hospital, Brasov 500326, Romania; Department of Interventional Cardiology, Brasov County Clinical Emergency Hospital, Brasov 500326, Romania

**Keywords:** dextrocardia, Bachmann bundle pacing, Left bundle branch pacing, Atrioventricular block, Case report

## Abstract

**Background:**

Conduction system pacing has been proven superior to conventional myocardial pacing in both atrial and ventricular conduction abnormalities. However, in dextrocardia, physiological pacing is difficult due to a lack of dedicated delivery tools. We present the case of a patient with dextrocardia and situs inversus, in whom both Bachmann bundle pacing and left bundle branch pacing were performed for advanced interatrial and atrioventricular block.

**Case summary:**

A 76-year-old patient with dextrocardia and complete situs inversus was admitted for recurrent syncope. The electrocardiogram showed advanced interatrial block and complete atrioventricular block. With no identifiable reversible factors for conduction abnormalities, we decided on complete atrioventricular physiological pacing. Using a modified 3D delivery catheter (inversion of the distal curve), we were able to reach both the interventricular and interatrial septum, where the leads were deployed. Conduction system potentials were recorded in the Bachmann and left bundle branch area, and conduction system capture was demonstrated using the current criteria. The pacing and sensing thresholds were optimal and stable at the 1-month follow-up.

**Discussion:**

Interatrial block and right appendage pacing are associated with a higher incidence of atrial fibrillation and adverse atrial remodelling, while long-term right ventricular pacing increases the risk for pacing-induced cardiomyopathy. By engaging the conduction system, physiological pacing leads to normal myocardial depolarization, thereby reducing the risk of the aforementioned adverse outcomes. Although atrial and ventricular conduction system pacing has been described in detail, this is the first case to illustrate these procedures in the setting of dextrocardia.

Learning points

- The current conduction system pacing catheters can be manually reshaped to reach the Bachmann bundle or the left bundle branch area in dextrocardia.
- Bachmann bundle pacing can correct advanced interatrial conduction delay and generate more physiologic P waves with proven electrophysiological capture criteria.
- Atrioventricular physiological pacing has the potential to correct interatrial, atrioventricular, and intraventricular conduction abnormalities within a single pacing procedure.

## Introduction

Permanent cardiac pacing is the only option for advanced atrioventricular block (AVB) to reduce mortality and morbidity. The modality of cardiac pacing evolved over the last decades from conventional right atrial (RA) and right ventricular (RV) direct myocardial capture to various forms of conduction system pacing.

Recent studies have shown the net benefit of left bundle branch pacing (LBBP) over RV pacing in terms of mortality and heart failure hospitalizations.^1^ The results of these studies have led to this technique being included in the recent cardiac pacing guidelines as an option for patients with advanced AVB and an expected high ventricular pacing burden.^2^

On the other hand, interatrial conduction block and conventional RA pacing were associated with an increased risk of atrial fibrillation occurrence and negative atrial remodeling.^3^ As a possible solution, Bachmann bundle pacing (BBP) was evaluated in several studies, showing promise in reducing the rate of these outcomes.^4^

For precise conduction system pacing, both the patient's anatomy and the pacing lead-delivery tools are crucial to a positive outcome. In this regard, dextrocardia poses a problem, as no available delivery catheters can reach the septum in this situation. Several case reports have presented a successful LBBP procedure, but the possibility of BBP has never been evaluated in patients with dextrocardia.^5^

We present the case of a patient with dextrocardia and situs inversus, in whom both BBP and LBBP were performed for advanced interatrial and AVB.

## Summary figure

**Figure:**
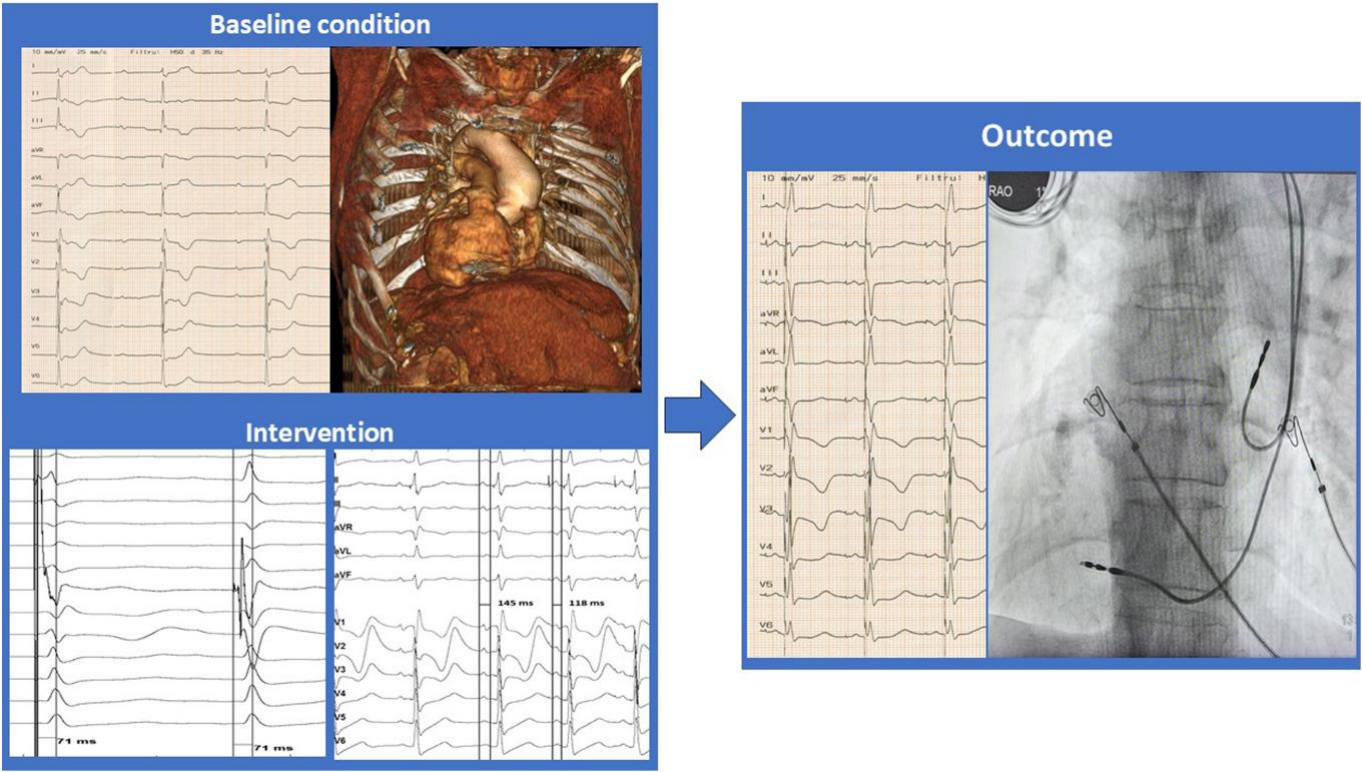


## Case summary

**Figure 1:**
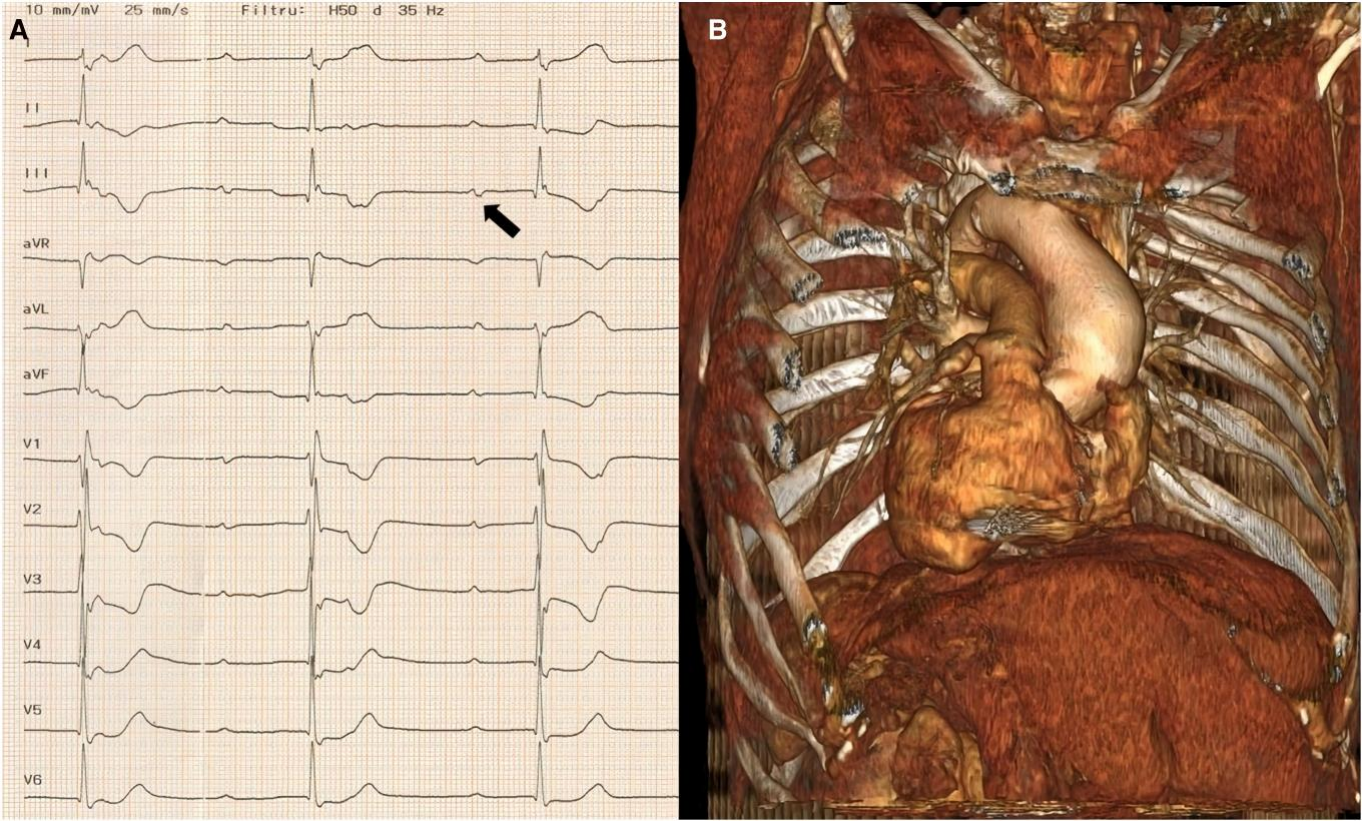
(*A*) presenting 12-lead ECG showing interatrial block (arrow) and complete AVB with RBBB escape rhythm; (*B*) 3D CT reconstruction showing dextrocardia and situs inversus. ECG, electrocardiogram; AVB, atrioventricular block; RBBB, right bundle branch block; CT, computed tomography.

A 76-year-old woman with known dextrocardia and situs inversus was brought to the emergency department for recurrent syncope in the context of complete AVB. Due to intermittent asystole, a temporary pacemaker was implanted in the emergency setting. The lab analysis showed a normal blood count and no significant pathological changes in the biochemistry tests, except for a slightly higher NT-proBNP value (650 pg/mL). The echocardiogram showed a normal left ventricle with preserved ejection fraction (60%), an enlarged left atrium (45 mm diameter), and mild mitral stenosis and regurgitation. The detailed evaluation of the electrocardiogram (ECG) recorded with a modified electrode position (electrodes placed on the opposite side of the midsternal line compared to standard placement) revealed a sinus rhythm with a prolonged P-wave duration (145 ms) and biphasic morphology in the inferior leads (especially DIII), suggesting advanced interatrial conduction block. There was a complete AVB with an escape rhythm with right bundle branch block morphology (*Figure 1A*). Due to a normal ejection fraction, the usual approach would have been RV pacing. But, with an anticipated high ventricular pacing burden, there was a subsequent risk of pacing-induced cardiomyopathy. Also, the presence of an interatrial block, which would be uncorrected by conventional RA appendage pacing, increases the risk of atrial fibrillation occurrence. In this context, we decided on a complete atrioventricular physiological pacing procedure to correct the interatrial conduction delay and to prevent pacing-induced cardiomyopathy in the future.

**Figure 2:**
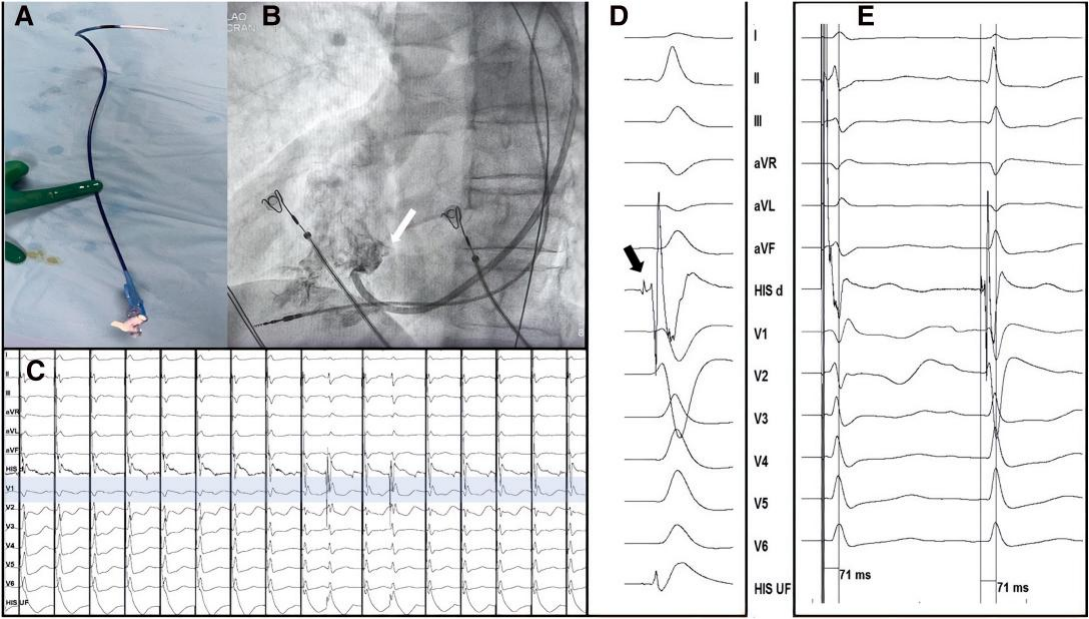
(*A*) modified ventricular delivery catheter with the distal curve pointing rightward; (*B*) contrast injection in the RV to delineate the tricuspid valve (white arrow). A temporary pacing lead is placed at the RV apex; (*C*) 12-lead ECG with continuous pacing during ventricular lead deployment showing a change in morphology from LBBB to RBBB in lead V1 once the left septal side has been reached (highlighted area); (*D*) The filtered intracardiac EGM (His d) shows a left bundle branch potential (black arrow); (*E*) Stimulus to R-wave peak interval equals potential to R-wave peak interval in lead V6 confirming left bundle capture. RV, right ventricle; ECG, electrocardiogram; LBBB, left bundle branch block; RBBB, right bundle branch block; EGM, electrogram.

**Figure 3:**
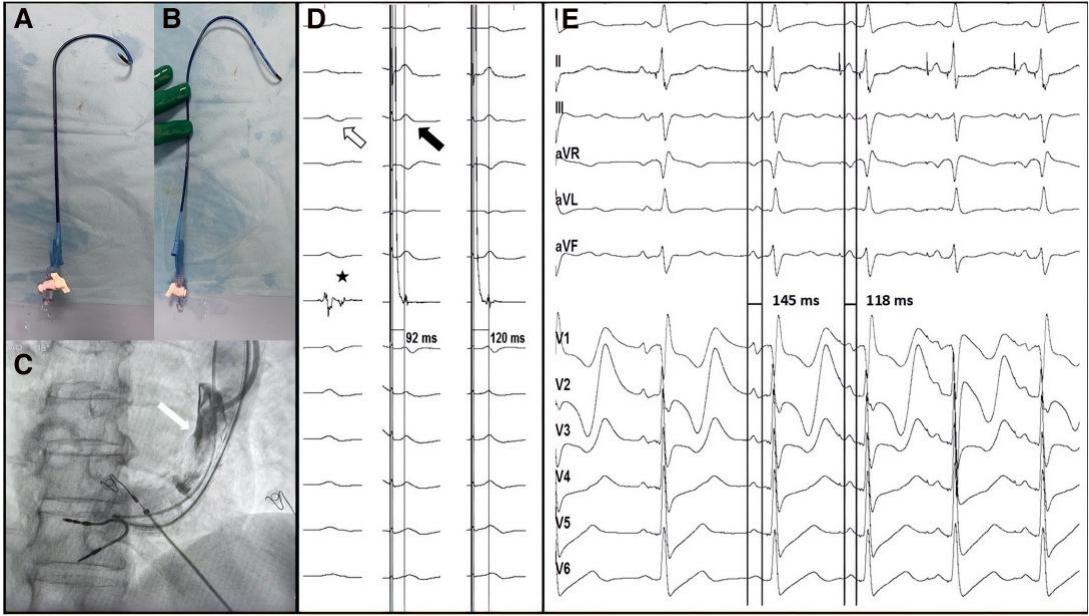
(*A*) initial atrial 3D delivery catheter with the distal curve pointing leftward; (*B*) modified atrial delivery catheter with the distal curve pointing rightward; (*C*) contrast injection in the distal superior vena cava to delineate the cavo-atrial junction (white arrow); (*D*) left panel: 12-lead P wave morphology showing interatrial block with biphasic waves in the inferior leads (hollow arrow). The intracardiac electrogram shows a split potential (asterisk), suggesting a Bachmann bundle potential. Middle panel: Non-selective Bachmann bundle capture at high amplitudes with a stimulus-to-electrogram duration of 92 msec and correction of interatrial block (black arrow). Right panel: Selective Bachmann bundle capture at lower amplitudes with a stimulus-to-electrogram duration of 120 msec; (*E*) 12-lead ECG showing a transition from sinus (first two P waves) to paced atrial rhythm (last three P waves). A significant reduction in P-wave duration from 145 to 118 msec was observed. ECG, electrocardiogram.

We performed a chest contrast CT to confirm the dextrocardia and the permeability of the superior venous system (*Figure 1B*). We chose the Biotronik Selectra 3D 55/42 catheter with the Solia S 60 lead for the ventricle and the Biotronik Selectra 3D 40/32 with the Solia S 53 lead for the atria (*Figure 3A*). Both catheters were manually reshaped to direct the distal curve towards the opposite site and reach the septum in the dextrocardia setting (*Figures 2A* and *3B*). Using a right axillary approach, we introduced the ventricular catheter beyond the tricuspid valve and injected a small amount of contrast to delineate it (*Figure 2B*). We advanced the delivery system approximately 1.5–2 cm from the tricuspid valve on the midseptum, where the paced QRS showed electrical discordance in the inferior leads. Fast lead rotations were applied until a narrow, paced QRS complex with a qR morphology in lead V1 was identified during continuous 12-lead ECG and electrogram monitoring, suggesting effective septal penetration (*Figure 2C*). A clear left bundle potential was recorded at this site with a potential to R wave peak time identical to the stimulus to R wave peak time in lead V6 (71 ms), confirming conduction system pacing (*Figure 2D* and *2E*). The ventricular pacing threshold was 1 V at 0.4 msec pulse duration with an R wave amplitude of 8.4 mV.

In the next step, we introduced the modified atrial catheter into the distal superior vena cava and injected a small amount of contrast to identify the cavoatrial junction (*Figure 3C*). We then placed the catheter-lead system in the high atrial septum, just below the junction. Using a combination of electrogram and pacemapping, we identified a spot with fragmented and split atrial signals, possibly suggesting a Bachmann bundle potential, where we recorded a narrow and tall paced P wave morphology (*Figure 3D*). After screwing the lead at that site, decremental pacing showed a clear transition between non-selective and selective BBP (with an isoelectric interval between the pacing artefact and P wave onset of 28 ms). The P wave had a significantly shorter duration (118 msec) and higher amplitude than in sinus rhythm, with correction of the interatrial block, as evidenced by the disappearance of the negative terminal part of the P wave in the inferior leads (*Figure 3D* and *3E*). The procedural pacing threshold was 1.2 V at a pulse duration of 0.4 msec, with a P-wave sensing of 1.4 mV. The pacemaker's algorithm for promoting atrial pacing was activated to fully benefit from the correction of interatrial block.

**Figure 4:**
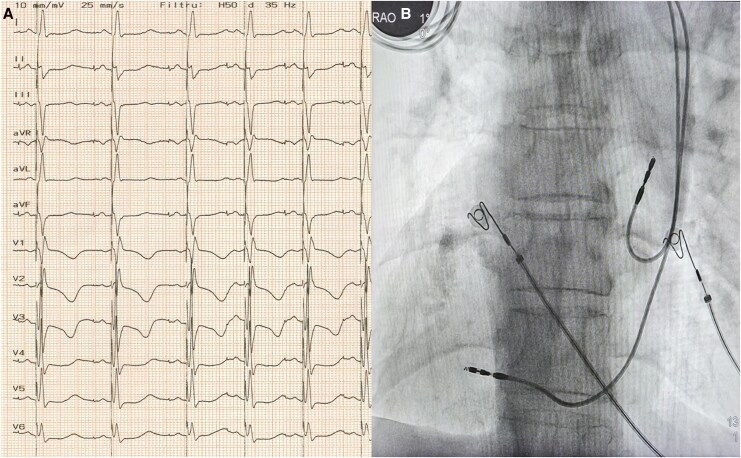
(*A*) final 12-lead ECG with paced atrial and ventricular rhythms; (*B*) posteroanterior fluoroscopic image with the final atrial and ventricular lead positions. ECG, electrocardiogram.

The patient was discharged the next day without any postprocedural complications (*Figure 4*). At the 1-month follow-up, the patient was clinically asymptomatic with stable pacemaker parameters (ventricular threshold of 0.75 V, R-wave sensing of 12.5 mV, atrial threshold of 1 V, and P-wave sensing of 1.4 mV), no atrial arrhythmias recorded and a normal ejection fraction (60%).

## Discussion

Dextrocardia is a rare congenital anomaly in which the heart is situated on the right side of the chest, with the apex pointing to the right. Its incidence ranges between 1 in 8.000 and 1 in 25.000 births and can be isolated or in the setting of situs inversus, where all the organs in the chest and abdomen are situated on the opposite side.^6^

If there are no accompanying malformations, dextrocardia is asymptomatic and does not require treatment.^7^ However, it is subject to the same risk of disease development as a normal heart, including electrical conduction abnormalities. In the latter situation, when pacemaker implantation is indicated, recent clinical evidence from randomized trials and multicentre registries has demonstrated the superiority of LBBP over conventional RV pacing for mortality and heart failure hospitalizations.^1,8^ However, dextrocardia poses a significant challenge for the physiological pacing procedure, as all the delivery tools are specifically designed for a normal heart position. To guide the lead across the septum, we selected the Selectra 3D catheter and manually reshaped it to mirror the original curve orientation and accommodate the heart's position. Due to the catheter's increased stiffness, we could achieve and maintain the new shape while manipulating it within the heart, ensuring it remained perpendicular to the septum. With the catheter`s support, the lead advanced easily through the midseptum, where we recorded a large left bundle signal, thereby achieving direct left bundle capture. This technique of catheter reshaping for LBBP in dextrocardia has been previously described in several case reports, utilizing both lumenless and stylet-driven leads.^9,10^

On the other hand, current data support a link between interatrial block, P-wave duration, and the risk of atrial fibrillation after initial ablation.^11^ To overcome this problem, recent studies have shown that BBP, intended to correct interatrial block, was associated with a significant reduction in atrial fibrillation rate and favourable atrial mechanics.^4,12^

Since the Bachmann bundle is located in the high interatrial septum, we faced the same challenge as delivering the ventricular lead. Therefore, we modified a Selectra 3D catheter with a smaller curve, allowing us to reverse the distal curve to reach the superior vena cava-RA junction. The commonly employed criteria for BBP are based on taller, narrower-paced P waves compared to sinus rhythm and the recording of a Bachmann bundle potential with non-selective to selective transitions during decremental pacing.^13^ All of these criteria were encountered in our patient. Also, with the limitation of a short follow-up, we confirmed the feasibility of this atrial lead location with respect to stability, pacing, and sensing thresholds.^4,14^

To the best of our knowledge, this was a rare instance of dextrocardia with AVB, in which both atrial and ventricular physiological pacing were successfully achieved using a modified delivery catheter.

## Conclusion

In patients with dextrocardia, interatrial and AVB can be successfully corrected with BBP and LBBP using a modified delivery catheter technique with optimal pacing parameters and lead stability.

## Lead author biography


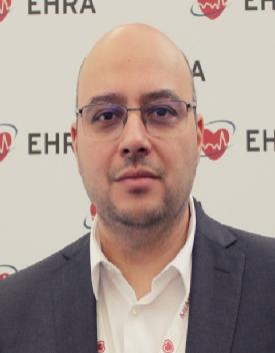


Dr. Catalin Pestrea is the Head of the Interventional Electrophysiology Unit of the Brasov County Emergency Clinical Hospital in Brasov, Romania. He specializes in interventional electrophysiology and cardiac device implantation, focusing on physiological pacing for bradycardia and cardiac resynchronization therapy indications.

## Data Availability

The data underlying this article are available in the article.
